# Arrhythmogenic cardiomyopathy: diagnosis, genetic background, and risk management

**DOI:** 10.1007/s12471-014-0563-7

**Published:** 2014-05-10

**Authors:** J. A. Groeneweg, J. F. van der Heijden, D. Dooijes, T. A. B. van Veen, J. P. van Tintelen, R. N. Hauer

**Affiliations:** 1 Department of Cardiology, University Medical Center Utrecht, HP Q05.2.314, Heidelberglaan 100, PO Box 85500, 3508 GA Utrecht, the Netherlands; 2 ICIN-Netherlands Heart Institute, Utrecht, the Netherlands; 3 Department of Medical Genetics, University Medical Center Utrecht, Utrecht, the Netherlands; 4 Department of Medical Physiology, University Medical Center Utrecht, Utrecht, the Netherlands; 5 Department of Genetics, University of Groningen, University Medical Center Groningen, Groningen, the Netherlands; 6 Durrer Center for Cardiogenetic Research, Utrecht, the Netherlands

**Keywords:** Arrhythmogenic right ventricular dysplasia/cardiomyopathy, Arrhythmogenic cardiomyopathy, Ventricular tachycardia, Sudden cardiac death, Genetics, Diagnosis

## Abstract

Arrhythmogenic cardiomyopathy (AC), also known as arrhythmogenic right ventricular dysplasia/cardiomyopathy (ARVD/C), is a hereditary disease characterised by ventricular arrhythmias, right ventricular and/or left ventricular dysfunction, and fibrofatty replacement of cardiomyocytes. Patients with AC typically present between the second and the fourth decade of life with ventricular tachycardias. However, sudden cardiac death (SCD) may be the first manifestation, often at young age in the concealed stage of disease. AC is diagnosed by a set of clinically applicable criteria defined by an international Task Force. The current Task Force Criteria are the essential standard for a correct diagnosis in individuals suspected of AC. The genetic substrate for AC is predominantly identified in genes encoding desmosomal proteins. In a minority of patients a non-desmosomal mutation predisposes to the phenotype. Risk stratification in AC is imperfect at present. Genotype-phenotype correlation analysis may provide more insight into risk profiles of index patients and family members. In addition to symptomatic treatment, prevention of SCD is the most important therapeutic goal in AC. Therapeutic options in symptomatic patients include antiarrhythmic drugs, catheter ablation, and ICD implantation. Furthermore, patients with AC and also all pathogenic mutation carriers should be advised against practising competitive and endurance sports.

## Introduction

Arrhythmogenic right ventricular (RV) dysplasia/cardiomyopathy (ARVD/C) is histopathologically characterised by progressive fibrofatty replacement of cardiomyocytes, primarily in the right ventricle [1–3]. However, histopathologically and functionally the left ventricle is affected in many cases and both ventricles are similarly affected by desmosomal and gap junctional protein redistribution [4, 5]. Because of these observations, at present arrhythmogenic cardiomyopathy (AC) is the preferred terminology [6]. AC can be defined as a structural myocardial disease *preceded* by ventricular arrhythmias. Typical ARVD/C with predominant RV abnormalities can be considered a large and important subcategory of AC. Clinical diagnosis is made according to international consensus-based Task Force Criteria [7, 8].

The first series of ARVD/C patients was published in 1982 [1]. It was described as a developmental disease of the RV musculature, hence the terminology ‘dysplasia’. In the past 25 years, increased insight into the development of the disease as well as the discovery of pathogenic gene mutations involved led to the current understanding that AC is a genetically determined cardiomyopathy. The molecular genetic substrate for the disease is mainly acknowledged in genes encoding desmosomal adhesion proteins in the intercalated disk [9–14].

This review provides an overview of AC, from phenotypic and genetic features of the disease, to diagnosis, risk stratification and treatment options.

## Epidemiology

Estimations of the prevalence of AC in the general population vary from 1:1000 to 1:5000 [15, 16]. The real prevalence of AC, however, is unknown and is presumably higher due to many non-diagnosed and misdiagnosed cases. In one study, features of AC were detected at post-mortem evaluation in as many as 20 % of sudden deaths occurring in people under 35 years of age [17]. In nearly half of them, no prior symptoms had been reported. Furthermore, in the study by Tabib et al. [18], 26 % of forensic autopsy cases following exercise-related sudden cardiac death (SCD) under the age of 30 years revealed AC.

From the genetic point of view, both men and women should be equally affected. However, men are more frequently diagnosed with AC than women. In a large multicentre study, 57 % of affected individuals were male. As many women as men show at least some signs of disease, but women less frequently fulfil criteria to meet the diagnosis [19]. It is speculated that (sports) activity or hormonal factors may play a role in this difference in severity of disease expression [20]. Familial disease, with AC diagnosis in at least one other family member besides the index patient, has been demonstrated in more than one-third of AC cases [21, 22].

## Presentation

**Fig. 1:**
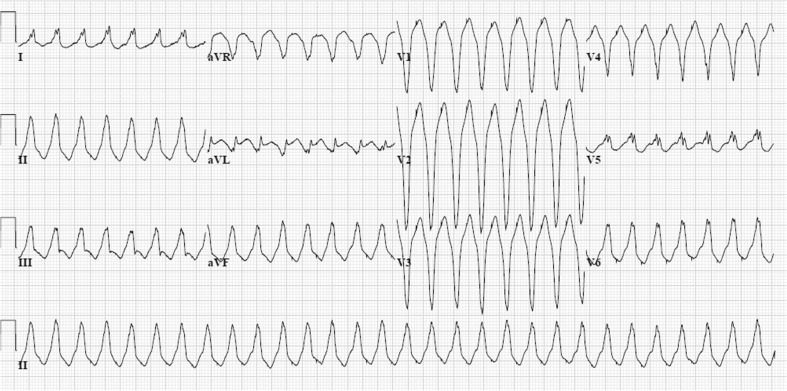
12-lead ECG of a ventricular tachycardia (VT) in an arrhythmogenic cardiomyopathy (AC) index patient with a pathogenic and a most likely pathogenic plakophilin-2 mutation (c.397C>T p.Gln133* and c.2615C>T p.Thr872Ile). The VT has a left bundle branch block morphology, with inferior axis. The QRS complex is predominantly negative in lead aVL and most positive in lead II, suggesting an origin from the right ventricular outflow tract area in the right ventricle

Patients with AC typically present between the second and the fourth decade of life with palpitations, lightheadedness, or syncope due to ventricular ectopy or (monomorphic) ventricular tachycardia (VT) with left bundle branch block (LBBB) morphology, thus originating from the right ventricle (Fig. 1). However, SCD may be the first clinical manifestation, often at young age in the concealed stage of disease. In a study by Quarta et al. [22], SCD was the presenting symptom in 50 % of index patients, with SCD occurring in 31 % at young age, i.e. between 14 and 20 years. A presentation with (aborted) SCD occurred less often in other studies [19, 23–25]. In the Dutch AC cohort, 2/149 (1.3 %) index patients presented with SCD and 8 % (12/149) with aborted SCD [21].

AC can present in four clinical stages which do not necessarily proceed from one into the other: 1) concealed stage without or with minimal structural disease, although SCD may occur, 2) overt stage with structural alterations of primarily the right ventricle, and episodes of monomorphic VT, 3) overt stage with obvious structural biventricular involvement, and 4) the end-stage of the disease with heart failure [1, 26, 27]. Recently, left ventricular (LV) dominant and primarily biventricular variants have also been described, in analogy with the AC concept [4, 28].

## Diagnosis

**Fig. 2:**
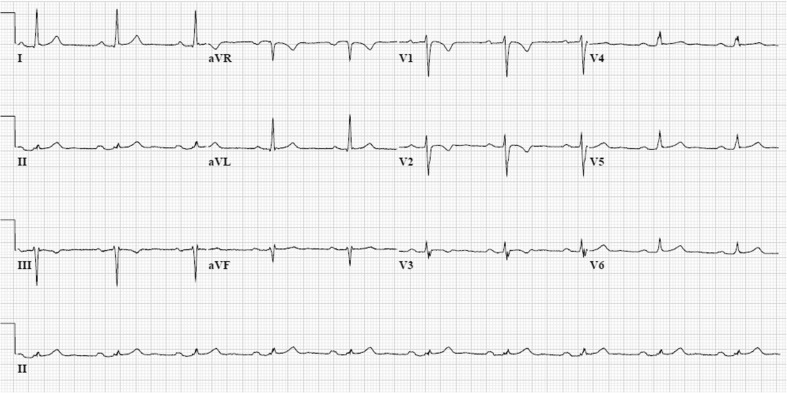
12-lead ECG (during atenolol 25 mg once daily) of the same index patient as in Figure 1. The ECG shows sinus rhythm, horizontal axis, and typical negative T waves in the right precordial leads V1-3. The terminal activation duration (from the nadir of the S wave to the end of all depolarisation deflections) in leads V1-3 is normal (≤55 ms)

Accurate AC diagnosis is critical due to lifelong implications, not only for the index patient but also for family members. Diagnosis has been facilitated by a set of clinically applicable criteria. These criteria, originally formulated for ARVD/C, were defined by an international Task Force based on consensus in 1994 and were revised in 2010 [7, 8]. In the revised Task Force Criteria (TFC) a minor criterion for (additional) LV involvement, inverted T waves in left precordial leads V4-6, was incorporated. The current TFC are the essential standard for a correct diagnosis in individuals suspected of AC. In addition, its universal acceptance contributes importantly to unambiguous interpretation of clinical studies and facilitates comparison of results. The TFC include six different categories: 1) global and/or regional dysfunction and structural RV alterations, 2) tissue characterisation, 3) depolarisation abnormalities, 4) repolarisation abnormalities, 5) arrhythmias, and 6) family history and genetics. Within these groups, diagnostic criteria are categorised as major or minor according to their disease specificity. A diagnosis of AC is made with the fulfilment of two major, one major and two minor, or with four minor TFC. From each different category, only one criterion can be counted for diagnosis, even when multiple criteria in one group are present. Table 1 provides an overview of the TFC defined in 2010. Figure 2 demonstrates typical repolarisation abnormalities in AC patients. Several studies suggested that this new set of criteria improved the diagnostic yield, with equal specificity [22, 29, 30]. Despite these efforts, AC diagnosis in the concealed stage of disease with subsequent risk for SCD still poses a great challenge for physicians.

**Table 1:** Overview of the current Task Force Criteria for arrhythmogenic cardiomyopathy (AC) diagnosis

I. Global or regional dysfunction and structural alterations	Major:
	- By 2D echo
	○ Regional RV akinesia, dyskinesia, or aneurysm
	○ And 1 of the following (end diastole): PLAX RVOT ≥32 mm (correct for body size [PLAX/BSA] ≥19 mm/m2), PSAX ≥36 mm (correct for body size [PSAX/BSA] ≥21 mm/m2, or fractional area change <33 %
	- By MRI
	○ Regional RV akinesia or dyskinesia or dyssynchronous RV contraction
	○ And 1 of the following: ratio of RVEDV to BSA ≥110 mL/m2 (male) or ≥100 mL/m2 (female), or RV ejection fraction ≤40 %
	- By RV cine-angiography
	○ Regional RV akinesia, dyskinesia, or aneurysm
	Minor:
	- By 2D echo
	○ Regional RV akinesia or dyskinesia
	○ And 1 of the following (end diastole): PLAX RVOT ≥29 to <32 mm (correct for body size [PLAX/BSA] ≥16 to <19 mm/m2), PSAX ≥32 to <36 mm (correct for body size [PSAX/BSA] ≥18 to <21 mm/m2, or fractional area change ≤33 to ≤40 %
	- By MRI
	○ Regional RV akinesia or dyskinesia or dyssynchronous RV contraction
	○ And 1 of the following: ratio of RVEDV to BSA ≥100 mL/m2 to <110 mL/m2 (male) or ≥90 mL/m2 to <100 mL/m2 (female), or RV ejection fraction >40 to ≤45 %
II. Tissue characterisation of wall	Major:
	- Residual myocytes <60 % by morphometric analysis (or <50 % if estimated), with fibrous replacement of the RV free wall myocardium in ≥1 sample, with or without fatty replacement of tissue on endomyocardial biopsy
	Minor:
	- Residual myocytes 60–75 % by morphometric analysis (or 50–65 % if estimated), with fibrous replacement of the RV free wall myocardium in ≥1 sample, with or without fatty replacement of tissue on endomyocardial biopsy
III. Repolarisation abnormalities	Major:
	- Inverted T waves in right precordial leads (V1, V2, V3) or beyond in individuals >14 years of age
	Minor:
	- Inverted T waves in leads V1 and V2 in individuals >14 years of age or in V4, V5, V6
	- Inverted T waves in leads V1, V2, V3 and V4 in individuals >14 years of age in the presence of complete right bundle branch block
IV. Depolarisation/conduction abnormalities	Major:
	- Epsilon wave (reproducible low-amplitude signals after the end of the QRS complex to onset of the T wave) in right precordial leads (V1, V2, V3)
	Minor:
	- Late potentials by SAECG in ≥1 of 3 parameters in the absence of a QRS duration of ≥110 ms on the standard ECG
	- Filtered QRS duration (fQRS) ≥114 ms
	- Duration of terminal QRS <40 uV (low-amplitude signal duration) ≥38 ms
	- Root-mean-square voltage of terminal 40 ms ≤20 uV
	- Terminal activation duration ≥55 ms measured from the nadir of the S wave to the end of all depolarisation deflections, including R′, in V1, V2 or V3 in the absence of complete right bundle branch block
V. Arrhythmias	Major:
	- Nonsustained or sustained ventricular tachycardia of left bundle branch morphology with superior axis (negative or indeterminate QRS in leads II, III, and aVF and positive in lead aVL)
	Minor:
	- Nonsustained or sustained ventricular tachycardia of RVOT configuration, left bundle branch block morphology with inferior axis (positive QRS in II, III and aVF and negative in aVL) or unknown axis
	- >500 ventricular extrasystoles per 24 h (Holter)
VI. Family history	Major:
	- AC confirmed in a first-degree relative who meets current TFC
	- AC confirmed pathologically at autopsy or surgery in a first-degree relative
	- Identification of a pathogenic mutation categorised as associated or probably associated with AC in the patient under evaluation
	Minor:
	- History of AC in a first-degree relative in whom it is not possible or practical to determine whether the family member meets current TFC
	- Premature sudden death (<35 years of age) due to suspected AC in a first-degree relative
	- AC confirmed pathologically or by current TFC in second-degree relative

The TFC, originally formulated for arrhythmogenic right ventricular dysplasia/cardiomyopathy (ARVD/C), include six different categories. Within these groups, diagnostic criteria are categorised as major or minor according to their disease specificity. A diagnosis of AC is made with the fulfilment of two major, one major and two minor, or with four minor TFC. From each different category, only one criterion can be counted for diagnosis, even when multiple criteria in one group are present

*BSA* body surface area, *first-degree family member* parent, sibling or child; *PLAX* parasternal long-axis view, *PSAX* parasternal short-axis view, *RVEDV* right ventricular end-diastolic volume, *RVOT* right ventricular outflow tract, *SAECG* signal averaged ECG, *second-degree family member* uncle/aunt, niece/nephew, grandparent

Specific evaluations are recommended in all patients suspected of AC: detailed history and family history, physical examination, 12-lead ECG (while off medications), signal averaged ECG, 24-hour Holter monitoring, maximal exercise testing, two-dimensional echocardiography with quantitative wall motion analysis, and more detailed imaging by cardiac magnetic resonance imaging (MRI) with delayed enhancement analysis. Invasive tests are also useful for diagnostic purposes: RV and LV cineangiography, electrophysiological testing, and endomyocardial biopsies for histopathological and immunohistochemical analysis [5, 31].

## Differential diagnosis

Early and occasionally late stages of AC may show similarities with a few other diseases. In particular, differentiation from idiopathic VT originating from the RV outflow tract (RVOT) can be challenging. However, idiopathic RVOT VT is a benign non-familial condition, in which the ECG shows no depolarisation or repolarisation abnormalities and no RV structural changes can be detected. Furthermore, VT episodes have a single morphology (LBBB morphology with inferior axis) and are based on abnormal automaticity or triggered activity, whereas reentry is the prevalent arrhythmia mechanism in AC [32, 33]. It is important to differentiate idiopathic RVOT VT from AC with regard to screening of family members, prognosis, and outcome of catheter ablation.

Another disease mimicking AC is cardiac sarcoidosis [34]. Clinical symptoms of cardiac involvement are present in about 5 % of all patients with sarcoidosis. In a study by Vasaiwala et al. [35] a remarkably high incidence (15 %) of cardiac sarcoidosis was found in patients with suspected AC. Presence of extracardiac sarcoidosis, mediastinal lymphadenopathy, septal conduction abnormalities, and septal scar on cardiac imaging may be indicative of cardiac sarcoidosis rather than AC as disease aetiology [36]. Histopathological findings from endomyocardial biopsies can differentiate between the two entities. Nonetheless, cardiac sarcoidosis may mimic AC even on the molecular level. In a recent study by Asimaki et al. [37] a markedly reduced immunoreactive signal in the intercalated disk of the desmosomal protein plakoglobin was observed in AC as well as in cardiac sarcoidosis patients.

Myocarditis might also be considered in the differential diagnosis. In general, endomyocardial biopsy is required to distinguish myocarditis from AC. In analogy with cardiac sarcoidosis, giant cell myocarditis may be indistinguishable from AC using immunohistochemical analysis. In contrast, viral myocarditis does not result in plakoglobin redistribution from the intercalated disk. Differential profiles of cytokines are implicated to underlie this difference in disruption of desmosomal proteins [37].

AC may also mimic dilated cardiomyopathy (DCM), especially in the more advanced stages of disease. However, patients with DCM usually present with heart failure rather than arrhythmias. Thus, patients with sustained VT or SCD as the initial symptom of a supposed DCM, should also be screened for AC.

Finally, there are a number of other rare differential diagnoses to consider: Brugada syndrome with similar electrocardiographic or RV arrhythmias, congenital abnormalities such as Uhl’s disease, Ebstein’s anomaly, and atrial-septal defects, RV infarction, and possibly pulmonary hypertension [27].

## Molecular genetic background of AC

In 2000, the seminal discovery of mutations in the plakoglobin (*JUP*) gene as the basis of Naxos disease, an autosomal recessive cardio-cutaneous syndrome with AC, directed the search for the genetic substrate to other genes encoding desmosomal proteins [9]. This candidate gene approach identified mutations first in the desmoplakin (*DSP*) gene, and thereafter in the plakophilin-2 (*PKP2*), desmoglein-2 (*DSG2*), and desmocollin-2 (*DSC2*) genes [10, 11, 13, 14].

In the Netherlands, as in most European countries, and in North America, mutations are predominantly found in the *PKP2* gene [21, 22, 25, 38, 39]. *PKP2* mutations are found in 52 % of Dutch AC index patients and even in 90 % of familial cases [21]. This high yield is partly explained by the occurrence of founder mutations in the Netherlands. Haplotype analysis suggested a founder effect of four different *PKP2* mutations [38, 40]. On the other hand, there are geographical differences in the prevalence of AC-related gene mutations [13, 41].

**Fig. 3:**
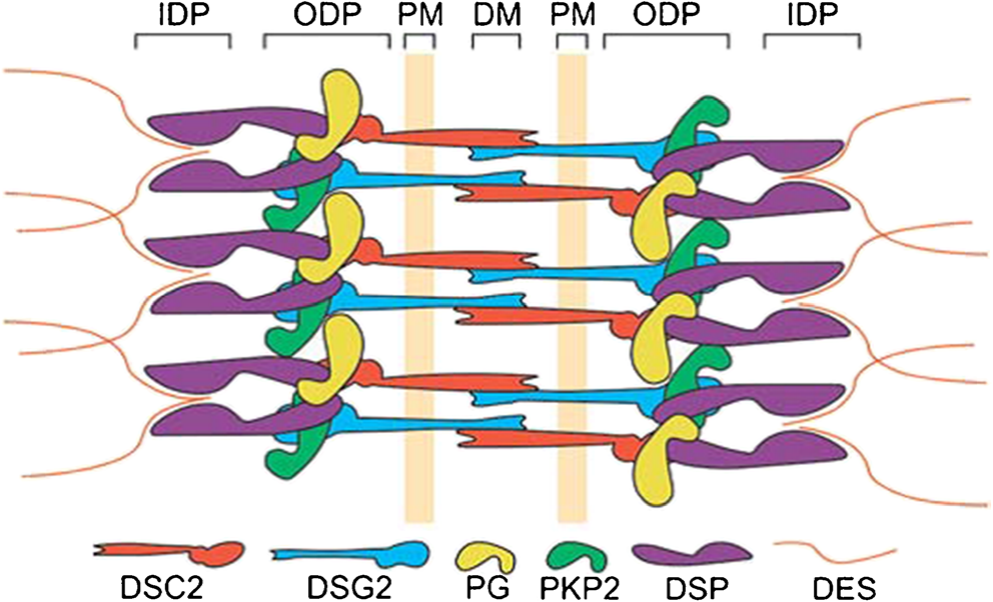
Schematic representation of the molecular organisation of cardiac desmosomes. The plasma membrane (*PM*) spanning proteins desmocollin-2 (*DSC2*) and desmoglein-2 (*DSG2*) interact in the extracellular space at the dense midline (*DM*). At the cytoplasmic side, they interact with plakoglobin (*PG*) and plakophilin-2 (*PKP2*) at the outer dense plaque (*ODP*). The PKP2 and PG also interact with desmoplakin (*DSP*). At the inner dense plaque (*IDP*), the C-terminus of DSP anchors the intermediate filament desmin (*DES*). (*Source*: Reprint with permission from: Van Tintelen et al. Curr Opin Cardiol. 2007;22:185–92)

Desmosomes are protein complexes located in the intercalated disk and are amongst others important for mechanical integrity of adjacent cardiomyocytes (Fig. 3) [42]. Desmosomal dysfunction due to a gene mutation may give rise to loss of mechanical cell-cell adhesion, and leads to down-regulation and/or altered distribution of other intercalated disk proteins, i.e. gap junction proteins (Connexin43) and sodium channels (Nav1.5) [43–45]. These alterations give rise to electrical cell-cell uncoupling and slow conduction, respectively, thereby providing a substrate for early activation delay resulting in ventricular tachyarrhythmia, a hallmark of AC [5, 12, 46, 47]. Presumably, at a later stage myocyte loss and fibrofatty replacement will have a major impact on tissue architecture, giving rise to zig-zag conduction pathways and load mismatch, further contributing to enhanced activation delay [32, 48].

Although the function of the desmosome and other components of the intercalated disk becomes increasingly clear, the exact mechanism by which a gene mutation results in the disease remains to be elucidated. Furthermore, not every subject with a mutation and thus a predisposition for AC develops signs and symptoms of the disease. Additional genetic factors, e.g. compound or digenic heterozygosity (carrying more than one mutation in one or more genes), or environmental factors such as exercise, may explain differences in severity of disease evolution within mutation carriers [49, 50].

**Fig. 4:**
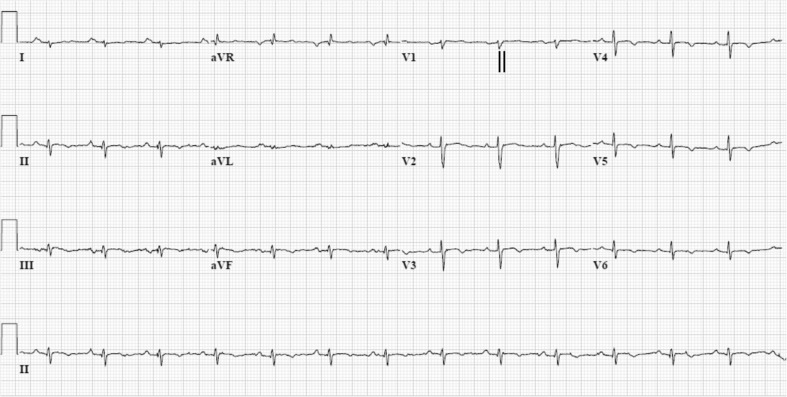
12-lead ECG (while off medications) of a phospholamban founder mutation carrier (c.40_42delAGA, p.Arg14del). The ECG shows sinus rhythm with right axis deviation, low voltages (<0.5 mV in standard leads), and characteristic negative T waves in left precordial leads from V3-6. The terminal activation duration (from the nadir of the S wave to the end of all depolarisation deflections) is 60 ms and therefore prolonged in lead V1 (*vertical black lines*)

In a minority of patients a non-desmosomal gene mutation is associated with the AC phenotype. Mutations in the non-desmosomal transforming growth factor β3 (TGFβ3), transmembrane protein 43 (*TMEM43*), desmin (*DES*), titin (*TTN*), lamin A/C (*LMNA*), αT-catenin (*CTNNA3*), and phospholamban (*PLN*) genes have been related to index patients and/or families with AC [51–57]. Of note, the *PLN* founder mutation c.40_42delAGA has been identified in 13 % of AC index patients in the Netherlands. AC patients with the *PLN* mutation often have low-voltage electrocardiograms, negative T waves in the left precordial leads V4-6, and additional LV involvement (Fig. 4) [58, 59]. To facilitate interpretation of genetic data, a large web-based database of genes and mutations underlying AC has been created (www.arvcdatabase.info). This database currently contains information on nearly 900 variants [60].

Not all forms of AC are genetically proven. These cases may be explained by mutations in yet unknown genes, by the contribution of genetic variants of unknown significance in the known genes, or epigenetic phenomena. The evaluation of a family history of AC, suggesting a still unknown genetic factor, is crucial in patients without identifiable genetic predisposition. Alternatively, these cases may be due to environmental factors, for example exercise. Exercise was a trigger and accelerator of the AC phenotype in a mouse model with JUP haploinsufficiency [61]. Very recently, the effect of exercise on AC disease expression was corroborated in humans. Of 87 pathogenic AC mutation carriers, 56 were endurance athletes. Endurance athletes were more likely to have symptoms at young age, fulfil the 2010 TFC, and had lower survival free from VT, ventricular fibrillation (VF), and heart failure. A reversible effect of reduction of sports activities, being a significant decrease in risk for VT or VF, was observed in those individuals who exercised the most (top quartile) [20].

## Risk stratification

Risk stratification in AC is imperfect at present. The annual mortality rates reported vary from 0.08–3.6 % [23, 24, 62, 63]. Retrospective analysis of clinical and pathological studies identified several risk factors for sudden death or appropriate ICD therapy, such as previously aborted SCD, syncope, young age, severe RV dysfunction, and LV involvement. Patients without VT had the best prognosis [23, 63]. Presence of sustained and non-sustained VT on Holter monitoring or during exercise testing, and sustained VT/VF during electrophysiological study were significant predictors of ICD therapy in a multivariate analysis in 84 patients treated with ICDs for primary prevention (appropriate ICD therapy in 48 %, of which 19 % for VF/ventricular flutter episodes) [64]. More recently, Te Riele et al. [65] showed that sustained arrhythmias in AC patients presenting alive seem to coincide with structural abnormalities and that patients with solely electrical abnormalities have a lower arrhythmic risk, implicating a role for RV structural abnormalities in risk stratification.

Molecular genetic analysis is also of importance in risk stratification in AC since cascade screening allows early detection of presymptomatic disease, identification of individuals at risk, and genetic counselling for this sudden death-predisposing disease. However, incorporating genetic results in AC risk stratification is hampered by incomplete penetrance and an extremely variable clinical expression. Mutation carriers may present with SCD but can also remain without signs and symptoms of the disease into old age. Therefore, genetic screening results should be viewed as probabilistic and as part of the overall clinical assessment.

Genotype-phenotype correlation analysis can provide more insight into risk profiles of index patients and family members. In contrast to index patients, mutation-positive family members have a better prognosis, with signs and symptoms of AC present in 50 % of relatives in their fifth decades of life [22]. Furthermore, family members with more than one genetic variant had a significant fivefold increase in risk of disease expression, suggesting gene-gene interactions and gene-dose effects. A genotype-phenotype correlation study by our group showed that compared with relatives of index patients without mutations, mutation carrying family members had 1) a sixfold higher risk of AC diagnosis, 2) a markedly enhanced risk for ventricular arrhythmias, and 3) earlier onset of AC signs and symptoms [21].

## Clinical management

In addition to symptomatic treatment, prevention of SCD is the most important therapeutic goal in AC. As there have been no randomised trials of AC treatment modalities, screening regimens, or medications, most recommendations are based on clinical expertise, results of retrospective registry-based studies, and studies on model systems.

Evidence suggests that, in the absence of sustained and non-sustained VT and/or high number of ventricular premature complexes (>1000 VPC/24 h), asymptomatic patients and healthy mutation carriers do not require treatment with an ICD for primary prevention [64]. These individuals should undergo regular cardiac evaluations (every 1–2 years) including 12-lead ECG, 24-h Holter monitoring, echocardiography, and exercise testing for timely identification of unfavourable signs necessitating ICD implantation.

However, in all patients diagnosed with or having signs or symptoms of AC as well as asymptomatic mutation carriers, specific lifestyle advice is recommended. Sports participation has been shown to increase the risk of SCD fivefold in AC patients [66]. Furthermore, excessive mechanical stress, such as during competitive sports activity and training, may aggravate underlying myocardial abnormalities and accelerate disease progression [20, 61]. Therefore, patients with AC, and in our opinion also all pathogenic mutation carriers, should be advised against practising competitive and endurance sports.

Therapeutic options in symptomatic patients with AC include antiarrhythmic drugs, catheter ablation, and ICD implantation. However, at present ICD implantation is the only proven lifesaving therapeutic modality for fast VT/VF. In patients who have presented with stable sustained VT but also patients and family members with non-sustained VT or >500 VPC on 24-h Holter monitoring, medication may be considered to reduce arrhythmias and symptoms. This pharmacological reduction may be critically important in combination with ICD therapy to reduce shock delivery. Since ventricular arrhythmias and cardiac arrest occur frequently during or after physical exercise or may be triggered by catecholamines, non-class III antiadrenergic beta-blockers are recommended. In the absence of adequate antiarrhythmic response, sotalol in an appropriate dose is the drug of first choice. Alternatively, amiodarone and flecainide have been reported to be useful [67]. Efficacy of drug treatment has to be evaluated by serial Holter monitoring and/or exercise testing.

Catheter ablation is an alternative in patients who are refractory to drug treatment and have frequent VT episodes (with a predominantly single morphology) [68]. Of note, the role of this therapy in AC patients is to improve quality of life by decreasing the frequency of episodes of sustained VT, symptomatic non-sustained VT, and ventricular ectopy. Accordingly, in a recent study by Philips et al. [69] the overall freedom from VT of 175 ablation procedures in 87 AC patients was 47 %, 21 %, and 15 %, at 1, 5, and 10 years, respectively, over a mean follow-up of 88.3 ± 66 months. The outcomes of VT ablation are improved with a combined endocardial and epicardial approach, incorporating a substrate-based strategy [69, 70].

Although antiarrhythmic drugs and catheter ablation may reduce VT burden, there is no proof from prospective trials that these therapies will also prevent SCD. Implantation of an ICD is indicated in AC patients who are intolerant to antiarrhythmic drug therapy and who are at serious risk for SCD, in patients with aborted cardiac arrest, intolerable fast VT and those with risk factors as mentioned above.

## AC in the Netherlands

In the Netherlands, a national collaboration of all University Medical Centers participating in ICIN has resulted in a large dataset with genetic and phenotypic characteristics of AC index patients and family members [21]. Since the prevalence of AC is relatively low, collaboration is essential for adequate studies and thereby improvement of insight into AC. This collaboration has expedited studies on AC-related genes, founder mutations, new diagnostic AC parameters, and clinical outcome of index patients and family members. Continuation of inclusion of more individuals, longer follow-up, and implementation of results of next generation DNA sequencing are needed to optimise further risk stratification.
